# In epithelial cancers, aberrant *COL17A1* promoter methylation predicts its misexpression and increased invasion

**DOI:** 10.1186/s13148-016-0290-6

**Published:** 2016-11-18

**Authors:** Pulari U. Thangavelu, Tibor Krenács, Eloise Dray, Pascal H. G. Duijf

**Affiliations:** 1 University of Queensland Diamantina Institute, The University of Queensland, Translational Research Institute, 37 Kent Street, Brisbane, QLD 4102 Australia; 2 1st Department of Pathology and Experimental Cancer Research, Semmelweis University and MTA-SE Cancer Progression Research Group, Budapest, Hungary; 3 Institute of Health and Biomedical Innovation, Queensland University of Technology, Translational Research Institute, Brisbane, QLD 4102 Australia

**Keywords:** Collagen XVII, Epigenetics, Invasion, Prognosis, Breast cancer, Cervical cancer

## Abstract

**Background:**

Metastasis is a leading cause of death among cancer patients. In the tumor microenvironment, altered levels of extracellular matrix proteins, such as collagens, can facilitate the first steps of cancer cell metastasis, including invasion into surrounding tissue and intravasation into the blood stream. However, the degree of misexpression of collagen genes in tumors remains understudied, even though this knowledge could greatly facilitate the development of cancer treatment options aimed at preventing metastasis.

**Methods:**

We systematically evaluate the expression of all 44 collagen genes in breast cancer and assess whether their misexpression provides clinical prognostic significance. We use immunohistochemistry on 150 ductal breast cancers and 361 cervical cancers and study DNA methylation in various epithelial cancers.

**Results:**

In breast cancer, various tests show that *COL4A1* and *COL4A2* overexpression and *COL17A1* (*BP180*, *BPAG2*) underexpression provide independent prognostic strength (HR = 1.25, 95% CI = 1.17–1.34, *p* = 3.03 × 10^−10^; HR = 1.18, 95% CI = 1.11–1.25, *p* = 8.11 × 10^−10^; HR = 0.86, 95% CI = 0.81–0.92, *p* = 4.57 × 10^−6^; respectively). Immunohistochemistry on ductal breast cancers confirmed that the *COL17A1* protein product, collagen XVII, is underexpressed. This strongly correlates with advanced stage, increased invasion, and postmenopausal status. In contrast, immunohistochemistry on cervical tumors showed that collagen XVII is overexpressed in cervical cancer and this is associated with increased local dissemination. Interestingly, consistent with the opposed direction of misexpression in these cancers, the *COL17A1* promoter is hypermethylated in breast cancer and hypomethylated in cervical cancer. We also find that the *COL17A1* promoter is hypomethylated in head and neck squamous cell carcinoma, lung squamous cell carcinoma, and lung adenocarcinoma, in all of which collagen XVII overexpression has previously been shown.

**Conclusions:**

Paradoxically, collagen XVII is underexpressed in breast cancer and overexpressed in cervical and other epithelial cancers. However, the *COL17A1* promoter methylation status accurately predicts both the direction of misexpression and the increased invasive nature for five out of five epithelial cancers. This implies that aberrant epigenetic control is a key driver of *COL17A1* gene misexpression and tumor cell invasion. These findings have significant clinical implications, suggesting that the *COL17A1* promoter methylation status can be used to predict patient outcome. Moreover, epigenetic targeting of *COL17A1* could represent a novel strategy to prevent metastasis in patients.

**Electronic supplementary material:**

The online version of this article (doi:10.1186/s13148-016-0290-6) contains supplementary material, which is available to authorized users.

## Background

Metastasis, the spread of cancer cells to distant organs, is one of the leading causes of death among cancer patients. To be able to disseminate, cancer cells need to overcome a number of barriers. In epithelia, cell-cell interactions and a basement membrane initially constitute major obstacles. In addition, once local invasion through the basement membrane has occurred, tumor cells need to be able to survive in the very different environment of the stroma [1].

The stroma consists of fibroblasts and extracellular matrix (ECM). The ECM is composed of polysaccharides, water, and stromal cell-secreted proteins, as well as soluble growth factors sequestered by matrix components [2]. Two types of macromolecules in the ECM, proteoglycans and fibrous proteins, influence cell growth, migration, attachment, and differentiation [3]. Collagens are fibrous proteins, which, with their high abundance in the ECM, contribute substantially to these processes [4]. In humans, a total of 44 collagen genes encode 28 varieties of collagen proteins [5].

While initially regarded as a physical barrier to tumor cell migration, recent studies have shown that collagens also support tumor progression depending on the stage of cancer development [6]. Associations between aberrant expression of collagens and tumor progression and metastasis are well established. For instance, increased density of collagen type I in lymph nodes is a clinical marker for breast cancer invasion [7]. Collagen I is also differentially expressed during colorectal tumorigenesis [8]. High levels of collagen type VI promote epithelial to mesenchymal transition, angiogenesis, inflammation, and chemotherapy resistance [9]. Collagen XI is expressed at high levels in human gliomas, colorectal cancer, and metastatic ovarian carcinoma, and at low levels in breast cancer [10–13]. Hence, collagen levels in the tumor stroma represent a valuable diagnostic parameter to differentiate between normal tissue, low-grade tumors, and metastatic cancer.

In contrast to other collagens, collagen types XIII, XVII, XXIII, and XXV are transmembrane proteins, characterized by an N-terminal cytoplasmic domain and an extracellular C-terminus that contains 3 to 15 collagenous domains [14]. Most research involving collagen XVII has focused on its role in healthy and diseased skin. Collagen XVII is a hemidesmosomal adhesion protein, whose expression in normal skin is limited to the basal keratinocytes, which are anchored to the basement membrane via collagen XVII [15]. However, it is overexpressed in squamous cell carcinoma (SCC) of the skin and in melanoma [15, 16].

Here, we systematically study the expression of all 44 collagen genes in breast cancer. We find that reduced expression of *COL17A1*, the gene that encodes collagen XVII, is most significantly associated with poor patient prognosis. Consistently, collagen XVII levels are reduced in breast tumors and this is strongly associated with tumor stage, invasion, and menopausal status. Conversely, collagen XVII levels are elevated in cervical cancer and this is associated with increased local metastasis. Interestingly, the *COL17A1* promoter methylation status correctly predicts the direction of collagen XVII misexpression in multiple types of epithelial cancers, including breast and cervical cancer.

## Results

### Underexpression of COL17A1 is a marker for poor prognosis in breast cancer

To identify collagens whose misexpression may contribute to breast cancer development, and in particular metastasis, we systematically evaluated expression levels of all 44 collagen genes. By combining microarray expression data from 26 previously published datasets, Cox proportional hazard analyses were performed based on expression level and distant metastasis-free survival (see the “Methods” section). For 18 of the 44 collagen genes, increased or decreased expression was significantly associated with poor patient outcome (HR with 95% CI <> 1, *p* < 0.05, 1052 < *n* < 4177; Additional file 1: Table S1).

We more stringently tested how well the misexpression of these genes might provide independent prognostic strength by including various other clinical parameters, such as lymph node status, tumor size, and menopausal status, all of which are included in Adjuvant! Online and the Nottingham Prognostic Index (NPI) [17, 18]. This reduced the number of significant associations from 18 to 8 collagen genes that passed all three tests with *p* < 0.05 (HR with 95% CI <> 1, 1052 < *n* < 4177) (Additional file 1: Table S1).

We also assessed whether patient survival significantly differed between patients whose tumors expressed low and high levels of these genes. This further reduced the number of genes to three, with overexpression of *COL4A1* and *COL4A2* and underexpression of *COL17A1* correlating with poor distant metastasis-free patient survival (*p* = 1.71 × 10^−5^, *n* = 3925; *p* = 0.0098, *n* = 4177; and *p* = 0.0001, *n* = 3925, respectively, log-rank test; Additional file 1: Table S1).

**Fig. 1:**
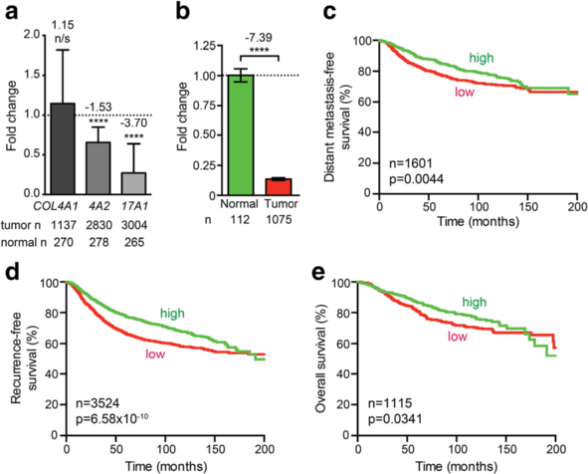
Reduced *COL17A1* expression correlates with poor breast cancer patient prognosis. **a** Fold change in normalized *COL4A1*, *COL4A2* (*4A2*), and *COL17A1* (*17A1*) expression using 28 combined previously published datasets. *p* values: *t* test. **b** Fold change in normalized *COL17A1* expression using the TCGA breast cancer RNAseq dataset. *p* value: Mann-Whitney *U* test. **c**–**e** Distant metastasis-free survival (**c**), recurrence-free survival (**d**), and overall survival (**e**) for breast cancer patients whose tumors express high (*green*) or low (*red*) *COL17A1* levels. Patients were split in low and high expression groups using the median expression level as the cut-off [23]. *p* values: log-rank test. *p* value summaries: *n/s* not significant; ****p < 0.0001

In an effort to independently validate these results, we used a combination of 28 other microarray datasets, as described [19, 20]. However, this showed that *COL4A1* expression did not significantly change (1.15-fold increase, *p* = 0.2574, *t* test, *n* = 1137) and that *COL4A2* expression was significantly reduced (1.53-fold decrease, *p* = 7.97 × 10^−10^, *n* = 2830), rather than increased (Fig. 1a). Only *COL17A1* misexpression was validated, as it consistently decreased in all analyses, the latter showing a 3.70-fold decrease (*p* = 4.93 × 10^−11^, *n* = 3004; Fig. 1a). Analysis of *COL17A1* levels using RNAseq data from The Cancer Genome Atlas (TCGA) [21, 22] also showed a significant downregulation (*p* < 0.0001, *n* = 1075; Fig. 1b). We therefore hereafter focus on *COL17A1*.

In addition to the above survival analyses (Additional file 1: Table S1), we investigated whether reduced *COL17A1* expression is associated with poor distant metastasis-free survival, recurrence-free survival, and overall patient survival, as previously described [23]. This confirmed significant correlations between these parameters (*p* = 0.0044, *n* = 1601; *p* = 6.58 × 10^−10^, *n* = 3524; *p* = 0.0341, *n* = 1115, respectively, log-rank test; Fig. 1c–e).

### Underexpression of collagen XVII is a marker for advanced stage, increased invasion, and postmenopausal status in breast cancer

**Fig. 2:**
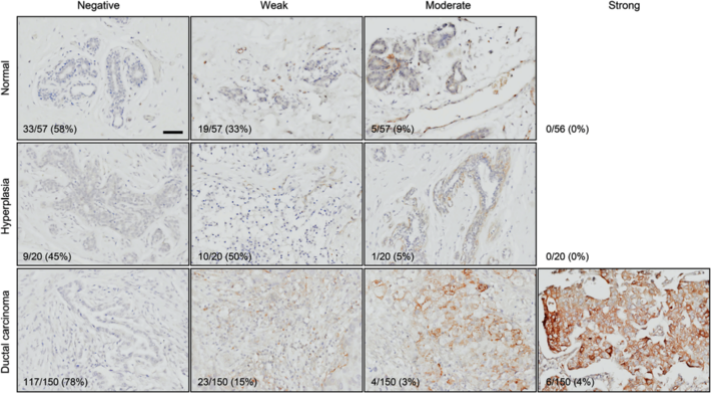
Collagen XVII is underexpressed in breast cancer. Tissue microarrays with a total of 57 normal breast tissues, 20 hyperplastic breast tissues, and 150 breast ductal carcinomas were stained with an anti-collagen XVII antibody [15, 16]. Numbers of samples in each category, as well as the total number of samples, are indicated. Percentages indicate frequencies of observations per row. *Scale bar*, 50 μm

Focusing on protein-level expression of collagen XVII, the product of the *COL17A1* gene, we next performed immunohistochemistry (IHC) using a previously well-characterized antibody [15, 16]. We used tissue microarrays with a total of 227 tissue samples, including 57 normal control breast samples, 20 hyperplastic breast samples, and 150 ductal breast carcinomas. The staining intensity ranged from negative to moderate in the normal and hyperplastic samples and from negative to strong in the tumor samples (Fig. 2). The corresponding H&E-stained images are available in Additional file 1: Figure S1. The increase in the fraction of strongly stained sections between normal and tumor samples (from 0% (0/56) to 4% (6/150), Fig. 2) was not statistically significant (*p* = 0.1908, Fisher’s exact test). Neither was the decrease in the fraction of moderately stained sections (from 9% (5/57) to 3% (4/150), *p* = 0.1185; Fig. 2). We therefore hereafter analyzed differences only based on whether staining was negative or positive.

Consistent with our above prognostic analyses, the ductal breast carcinomas stained positive for collagen XVII significantly less frequently that normal control tissue (22% (33/150) vs 42% (24/57), *p* = 0.0038, Fisher’s exact test; Table 1). The frequency in hyperplastic mammary epithelium, potentially a precursor of mammary carcinoma, did not significantly differ (55% (11/20) vs 42% (24/57), *p* = 0.2308).

**Table 1:** Collagen XVII α1 expression in normal, hyperplastic, and tumor breast tissue

Variable	Number positive/total (%)	*p* value vs normal^a,b^	Other (*p* value)^a,b^
All samples			
Normal breast	24/57 (42)		
Hyperplasia	11/20 (55)	0.2308	
Ductal carcinoma	33/150 (22)	*0.0038*	
Grade			
1	4/34 (12)	*0.0019*	
2	25/102 (25)	*0.0174*	
3	2/10 (20)	0.1665	
Stage			
I	0/6 (0)	0.0480	I/II vs III/VI (0.0287)
II	29/105 (28)	0.0452	
I/II	29/111 (26)	0.0273	
III	4/33 (12)	*0.0024*	
IV	0/6 (0)	0.0480	
III/IV	4/39 (10)	*0.0005*	
Tumor invasion			
T1	2/8 (25)	0.3015	T1/2 vs T3/4 (*0.0143*)
T2	25/89 (28)	0.0587	
T1/T2	27/97 (28)	0.0512	
T3	4/28 (14)	*0.0084*	
T4	2/25 (8)	*0.0015*	
T3/T4	6/53 (11)	*0.0002*	
Nodal status			
N0	25/98 (26)	0.0254	0 vs 1 (0.1625)
N1	6/38 (16)	*0.0057*	0/1 vs 2/3 (0.3657)
N2	2/12 (17)	0.0894	
N3	0/2 (0)	0.3477	
Metastasis			
M0	33/146 (23)	*0.0052*	M0 vs M1 (0.3659)
M1	0/4 (0)	0.1266	
Estrogen receptor status			
ER+	13/63 (21)	*0.0093*	ER+ vs ER− (0.5000)
ER−	15/78 (19)	*0.0035*	
Progesterone receptor status			
PR+	11/44 (25)	0.0562	PR+ vs PR− (0.2177)
PR−	17/96 (18)	*0.0011*	
HER2 status			
HER2-	4/32 (13)	*0.0031*	HER2− vs HER2+ (0.1702)
HER2+	24/108 (22)	*0.0068*	
Age/menopausal status			
<48	21/71 (30)	0.0988	<48 vs ≥48 (0.0268)
48–54	6/37 (16)	*0.0040*	<51 vs ≥51 (*0.0218*)
>54	6/42 (14)	*0.0024*	<54 vs ≥54 (0.0480)
			<48 vs ≥54 (0.0263)

^**a**^Calculated by Fisher’s exact test

^**b**^ *p* values in italic font remain statistically significant after accounting for multiple testing using a false discovery rate (FDR) of 5% [40]

We next assessed whether the reduced frequency of collagen XVII-positive staining in the tumors was specifically associated with tumor grade and markers used for diagnosis and determining the most effective treatment regimen. We did not observe any remarkable differences between tumors with differential estrogen receptor (ER), progesterone receptor (PR), or HER2 amplification status (Table 1). However, the already significant reduction in collagen XVII positivity from 42% (24/57) in normal samples to 26% (29/111) in early stage tumors (stage I/II; *p* = 0.0273) was further reduced significantly to 10% (4/39) in late stage cancers (stages III/IV; *p* = 0.0005 compared to normal, *p* = 0.0287 compared to stages I/II). The fractions of tumors that stained positive also significantly declined as tumors become more invasive, from 28% (27/97) in tumors that only locally invaded the submucosa and/or muscle (T1/2) to 11% (6/53) in tumors that invaded through underlying muscle and/or into other organs (T3/4) (*p* = 0.0143). Only 8% (2/25) of the most invasive cancers stained positive (*p* = 0.0015 compared to 42% (24/57) in normal tissue). Collagen XVII positivity also reduced with an increase in the number of positive lymph nodes and metastasis. Yet, this trend was not statistically significant (Table 1). Finally, with positive staining of 30% (21/71) of premenopausal carcinomas and 14% (4/42) of postmenopausal samples, menopausal status had a strong impact on collagen XVII expression (*p* = 0.0263; Table 1). Taken together, we conclude that the frequency of collagen XVII-positive tumors declines with advanced stage, increased invasion, and postmenopause.

### Collagen XVII is overexpressed in cervical cancer

**Fig. 3:**
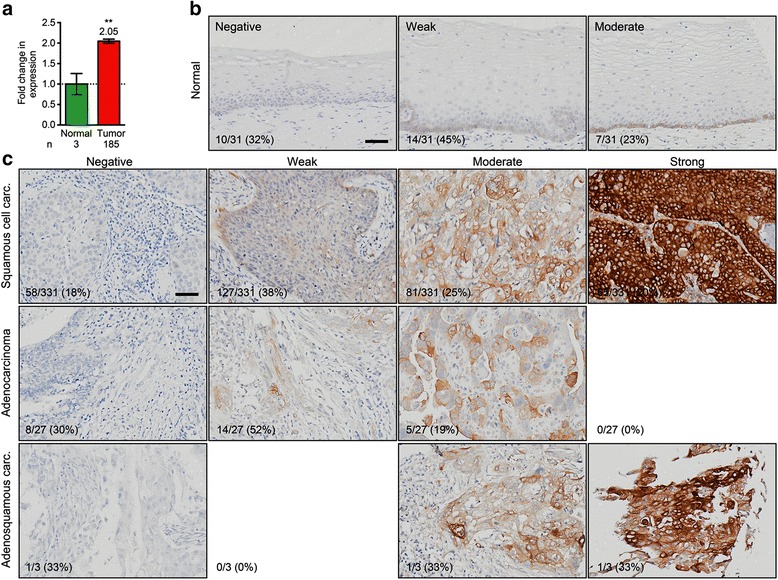
Collagen XVII is overexpressed in cervical cancer. **a** *COL17A1* is overexpressed twofold in the TCGA cervical cancer dataset. *p* values: *t* test. **b**, **c** Tissue microarrays with 31 normal control cervical tissue samples (**b**) and 331 cervical squamous cell carcinomas, 27 cervical adenocarcinomas, and 3 cervical adenosquamous carcinomas (**c**) were stained with an anti-collagen XVII antibody. Numbers of samples in each category, as well as the total number of samples, are indicated. Percentages indicate frequencies of observations per row. *Scale bar*, 50 μm

Our interest in women’s cancers prompted us to also assess collagen XVII expression in cervical cancer. In contrast to breast tumors, cervical cancers show a significant twofold increase in COL17A1 mRNA level compared to normal control tissue (*p* = 0.0046, Mann-Whitney *U* test, *n* = 185; Fig. 3a). We next performed IHC on tissue microarrays with 31 normal control tissues, 331 squamous cell carcinomas (SCC), 27 adenocarcinomas, and 3 adenosquamous carcinomas. Normal cervix typically stains weakly positive for collagen XVII (Fig. 3b). In contrast, among cervical SCCs, collagen XVII staining ranges from negative to strong. In addition, when positive, collagen XVII expression is observed in a much larger proportion of the cells as compared to normal tissue (Fig. 3c). In comparison to normal cervix, a significantly higher fraction of SCCs stains positive (68% (21/31) and 82% (273/331), respectively; *p* = 0.0442, Fisher’s exact test), moderately to strongly positive (23% (7/31) and 44% (146/331), respectively; *p* = 0.0146), or strongly positive (0% (0/31) and 20% (65/331), respectively; *p* = 0.0016) (Table 2). Among cervical adenocarcinomas and adenosquamous carcinomas, the fractions of positive and strongly positive samples were also increased, respectively. However, these increases were not statistically significant (Table 2).

**Table 2:** Collagen XVII α1 expression in normal cervix and cervical tumor tissues

Tissue	Total number of samples	Positive (%)	*p* value vs normal^a,b^	Moderate or strong (%)	*p* value vs normal^a,b^	Strong (%)	*p* value vs normal^a,b^
Normal cervix	31	21 (68)		7 (23)		0 (0)	
Squamous cell carcinoma	331	273 (82)	*0.0442*	146 (44)	*0.0146*	65 (20)	*0.0016*
Adenocarcinoma	27	19 (70)	0.5283	5 (19)	0.4769	0 (0)	1.0000
Adenosquamous carcinoma	3	2 (67)	0.7040	2 (67)	0.1644	1 (33)	0.0882

^**a**^Calculated by Fisher’s exact test

^**b**^ *p* values in italic font remain statistically significant after accounting for multiple testing using a false discovery rate (FDR) of 5% [40]

### Collagen XVII overexpression is a marker for local metastasis in cervical cancer

We also investigated collagen XVII staining in relation to lymph node status and distant metastasis. Only two of the patients were diagnosed with distant metastasis, thus preventing us from evaluating this parameter. However, among the three cancer types, the fraction of tumors that stained positive for collagen XVII in lymph node-positive cervical cancers was consistently higher than that fraction in lymph node-negative tumors (Table 3). Overall, increased collagen XVII expression is significantly associated with an increase in local metastasis (81% (258/320) vs 94% (33/35); *p* = 0.0293, Fisher’s exact test; Table 3).

**Table 3:** Collagen XVII α1 expression in relation to lymph node status in cervical cancer

Variable	Total	Lymph node-negative:	Lymph node-positive:	*p* value^a^
		Stained positive (%)	Stained positive (%)	
Squamous cell carcinoma	325	242/295 (82)	28/30 (93)	0.0860
Adenocarcinoma	27	16/24 (67)	3/3 (100)	0.3313
Adenosquamous carcinoma	3	0/1 (0)	2/2 (100)	0.3333
All cancers	355	258/320 (81)	33/35 (94)	0.0293

^**a**^Calculated by Fisher’s exact test

### In breast cancer, the COL17A1 promoter is hypermethylated and this correlates with reduced gene expression

**Fig. 4:**
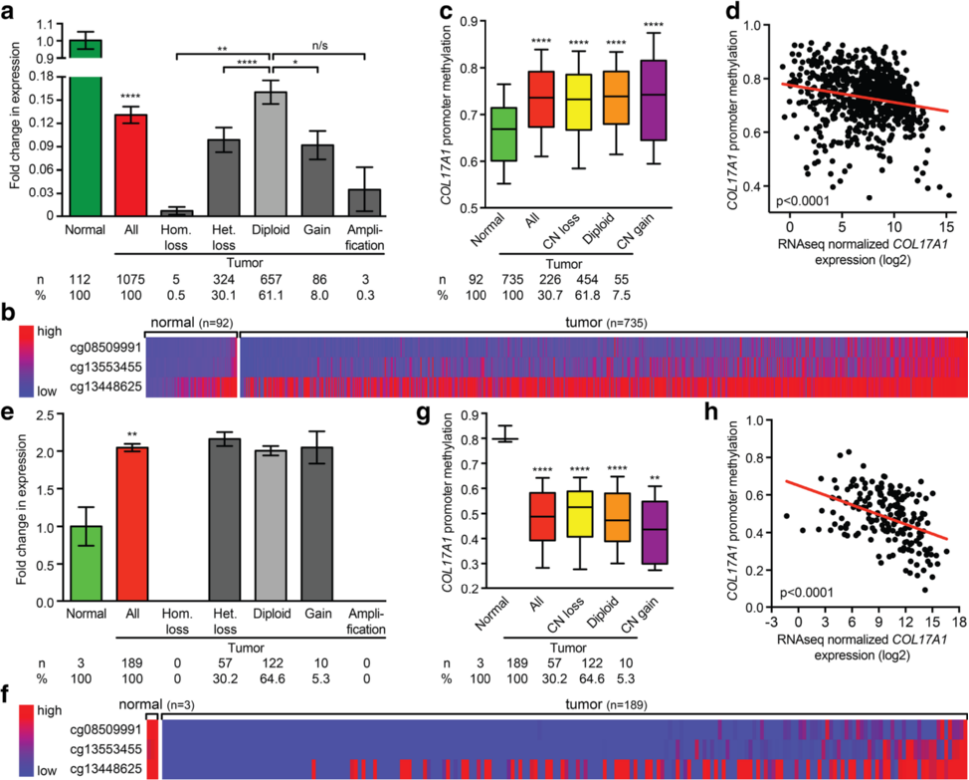
The *COL17A1* promoter methylation status predicts the direction of misexpression in breast and cervical cancer. **a** *COL17A1* allelic copy number gains and losses in relation to normalized *COL17A1* expression level. Data are extracted from TCGA breast cancer RNAseq V2 RSEM and SNP6 array GISTIC copy number datasets. *Error bars* represent standard error of the mean. *p* values: Mann-Whitney *U* test. **b** Heat map of the degree of promoter methylation based on *β* values for each indicated probe. Each three-probe column corresponds to a sample. Data were extracted from the TCGA Illumina Infinium HumanMethylation450 breast cancer dataset [21, 22]. **c** Box plot for *COL17A1* promoter methylation comparing normal samples to all samples, as well as to samples with indicated allelic copy numbers. *Whiskers* represent 10–90 percentiles of the data. *p* values: Mann-Whitney *U* test. **d** Scatter plot of *COL17A1* promoter methylation compared to normalized *COL17A1* gene expression. *p* value for linear regression line: Spearman correlation. **e**–**h** Graphs as in (**a**–**d**), respectively, for cervical cancer. Data were derived from the TCGA cervical dataset. *p* value summaries: *n/s* not significant; *p < 0.05; **p < 0.01; ****p < 0.0001

The misexpression of collagen XVII in breast and cervical cancers in opposite directions, both at mRNA and at protein levels, prompted us to investigate whether changes in allelic copy numbers could provide an explanation for this. We used SNP array GISTIC copy number data and RNAseq data from the respective TCGA studies [21, 22]. While the majority of the breast cancers (61% (657/1075)) had retained two copies of the *COL17A1* locus, nearly a third, 31% (329/1075), lost one or both alleles. This led to a significant reduction of *COL17A1* mRNA levels compared to diploid tumors (*p* < 0.0001, Mann-Whitney *U* test), whose levels were already significantly lower than in normal breast tissue (*n* = 112; *p* < 0.0001; Fig. 4a). Interestingly, despite the fact that the remaining 8.3% (89/1075) of the tumors had gained extra copies of the *COL17A1* locus, their mRNA levels were significantly lower than those in diploid cancers (*p* = 0.0218).

The observation that copy number changes do not have a major impact on *COL17A1* expression levels in breast cancer suggests that additional mechanisms regulate this gene’s expression. Tumor cells may silence gene expression through promoter hypermethylation [24]. Hence, we compared the *COL17A1* promoter methylation status in normal breast and breast cancer samples using TCGA DNA methylation data [21, 22] (Additional file 1: Figure S2 and Additional file 2). This revealed that the *COL17A1* promoter is indeed hypermethylated in breast tumors (*n* = 735 tumors, *n* = 92 normal samples; *p* < 0.0001, Mann-Whitney *U* test; Fig. 4b, c) and this hypermethylation is independent of *COL17A1* allelic copy number variations (*p* < 0.0001; Fig. 4c). In addition, there is a strong negative correlation between *COL17A1* promoter methylation and gene expression (Spearman *p* < 0.0001; Fig. 4d). Taken together, these analyses suggest that reduced *COL17A1* expression in breast cancer is caused by hypermethylation of the *COL17A1* promoter.

### In cervical cancer, the COL17A1 promoter is hypomethylated and this correlates with increased gene expression

In cervical cancer, the *COL17A1* locus was less frequently subject to copy number changes than in breast cancer (Fig. 4e). When copy number alterations did occur, this did not affect its expression (Fig. 4e). In contrast, the *COL17A1* promoter was considerably hypomethylated compared to normal tissue (*n* = 189 tumors, *n* = 3 normal samples; *p* < 0.0001, Mann-Whitney *U* test; Fig. 4f, g). This occurred irrespective of allelic copy number changes (*p* ≤ 0.0035; Fig. 4g), and *COL17A1* expression strongly correlated inversely with the degree of methylation of its promoter (Spearman *p* < 0.0001; Fig. 4h). These data strongly suggest that increased *COL17A1* expression in cervical cancer is caused by hypomethylation of the *COL17A1* promoter.

### The COL17A1 promoter methylation status accurately predicts the direction of misexpression in epithelial cancers

**Fig. 5:**
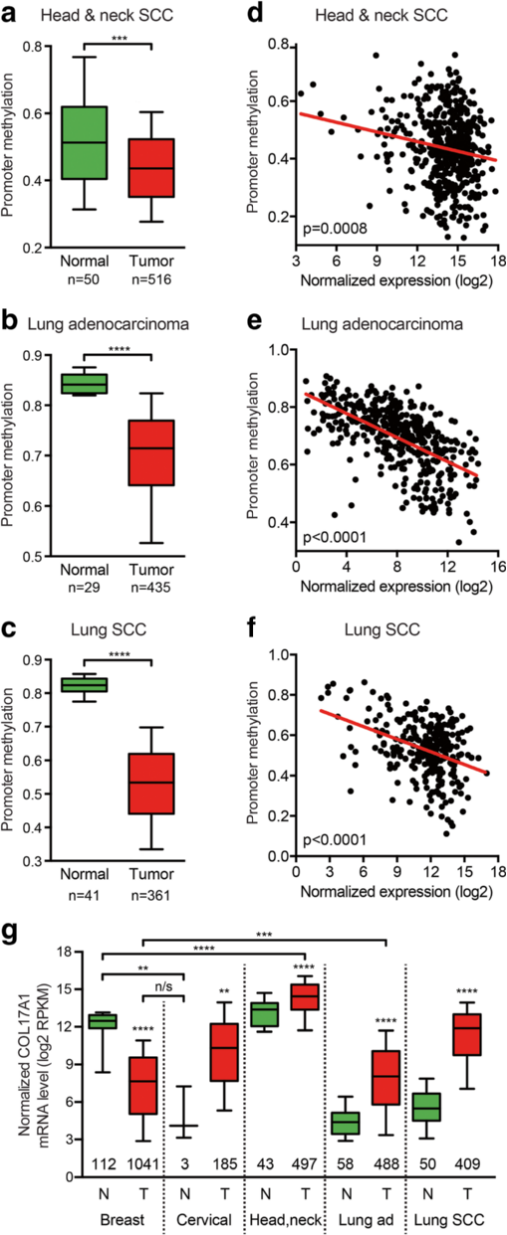
The *COL17A1* promoter is hypomethylated in head and neck and lung cancers. **a**–**c** Methylation status of the *COL17A1* promoter in head and neck squamous cell carcinoma (SCC), lung adenocarcinoma, and lung SCC using the respective TCGA datasets [41–43], as in Fig. 4c. *p* values: Mann-Whitney *U* test. **d**–**f** Scatter plots of *COL17A1* promoter methylation compared to normalized gene expression for indicated cancer types, as in Fig. 4d. *p* values: Spearman correlation for linear regression. **g** Box plot comparing the absolute COL17A1 mRNA levels in five epithelial tumor types and their respective matched normal control tissues. Data were extracted from TCGA RNAseq datasets [21, 22, 41–43]. *N* normal tissues, *T* tumor tissues. Sample numbers are indicated below each box. *p* values: Mann-Whitney *U* tests. *p* value summaries: **p < 0.01; ***p < 0.001; ****p < 0.0001

Interestingly, collagen XVII overexpression is observed by IHC in various other cancers, including skin SCC, melanoma, non-small cell lung cancer, lung adenocarcinoma, lung SCC, and head and neck SCC [15, 16, 25–28]. This led us to also investigate the promoter methylation status for these cancers. For the former three, TCGA data were either not available or the low number of normal samples (*n* = 2) precluded accurate analysis. For the remaining cancers, we find that the degree of *COL17A1* promoter methylation is significantly reduced in the tumor samples (*n* = 516, *n* = 435, *n* = 361, respectively) compared to normal control samples (*n* = 50, *n* = 29, *n* = 41, respectively; *p* ≤ 0.0002, Mann-Whitney *U* test; Fig. 5a–c). In addition, similar to breast and cervical cancer (Fig. 4d, h), the degree of promoter methylation highly significantly correlates inversely with gene expression for these cancers (*p* ≤ 0.0008, Spearman correlation; Fig. 5d–f). Thus, the *COL17A1* promoter methylation status accurately predicts the direction of the misexpression of collagen XVII in five out of five cancer types. This indicates that the differential *COL17A1* promoter methylation dictates whether collagen XVII is under- or overexpressed in these epithelial cancers (*p* = 0.0313, binomial test).

### Absolute levels of COL17A1 expression differ between normal and tumor tissues of different origin

We observed that COL17A1 is underexpressed in breast cancer and overexpressed in cervical and other epithelial cancers. This raises the possibility that the absolute COL17A1 levels are similar in different cancers while the basal COL17A1 levels are high in normal breast tissue and low in other normal epithelia. To test this hypothesis, we directly compared the absolute COL17A1 mRNA levels in the five tumor types and the respective matched normal control tissues investigated above (Fig. 5g). This scenario seemed to apply when we compared breast to lung. Specifically, COL17A1 levels in breast carcinomas and lung adenocarcinomas are similar, while the COL17A1 levels are highest in normal breast tissue and lowest in normal lung tissue (Fig. 5g). Generally, however, this is not the case. For instance, head and neck SCCs express significantly higher levels than normal breast tissue (*p* < 0.0001, Mann-Whitney *U* test; *n* = 497 and *n* = 112, respectively). Also, in normal cervix, COL17A1 levels do not significantly differ from those in breast carcinoma (*p* = 0.1417, Mann-Whitney *U* test; *n* = 3 and *n* = 1041, respectively), whereas the levels in normal cervix are significantly lower than in normal breast tissue (*p* = 0.0027, Mann-Whitney *U* test; *n* = 3 and *n* = 112, respectively; Fig. 5g). Thus, while COL17A1 misexpression is common in epithelial cancers, the absolute COL17A1 levels vary widely between and among tumors and matched normal samples of different tissue origin.

## Discussion

We systematically assessed the potential involvement of all 44 collagens in breast cancer progression and metastasis. This analysis identified overexpression of *COL4A1* and *COL4A2* as strong independent markers. However, independent assessment using TCGA RNAseq data did not validate this observation. At the protein level, a number of collagens, including collagens I, III, IV-α1, IV-α2, and V, are differentially expressed in breast cancer [29, 30]. This seeming discrepancy could be explained by the fact that our assessment is based on mRNA rather than on protein levels. Alternatively, sample-averaged mRNA levels could mask important differences in the local distribution of collagens within tumors, as observed for collagens IV, XV, and XIX in invasive ductal carcinomas [31]. Nevertheless, our approach led us to identify reduced *COL17A1* mRNA levels as a strong independent prognostic marker in breast cancer development and this was not only sustained at the protein level, but it was also strongly associated with advanced stage, increased invasion, and postmenopausal status.

We find that collagen XVII is underexpressed in breast cancer, while it is overexpressed in various other cancers, including cervical cancer, as we show here, and skin SCC, melanoma, head and neck SCC, non-small cell lung cancer, lung adenocarcinoma, and lung SCC, as previously described [15, 16, 25–28]. The cancer type-specific misexpression in opposing directions is not unique for collagen XVII. Collagen XI is expressed at high levels in human gliomas [10], colorectal cancer [11], and ovarian carcinoma [12] but at low levels in breast cancer [13]. Also, collagen XI protein-level expression positively correlates with ovarian cancer metastasis, but it inversely correlates with breast cancer metastasis [12, 13].

It is well established that differential DNA methylation in promoter regions causes misexpression of genes in cancer [32]. Collagen gene expression is altered in this manner in various cancer types [33–35]. However, to our knowledge, this is the first study that links the cancer type-specific, opposed direction of the misexpression of any collagen gene to cancer type-specific epigenetic alterations. It would be interesting to see if differential promoter methylation of other collagen genes, such as those encoding collagens XI-α1 and XI-α2 [10–13], could similarly explain opposed misexpression in distinct cancer types.

While consistent with epigenetic alterations, it remains paradoxical that both reduced collagen XVII expression in breast cancer and increased collagen XVII expression in other cancers are associated with increased tumor cell invasion and metastasis [15, 16, 25–28]. We investigated the possibility that the absolute COL17A1 levels are similar in different cancers while the normal COL17A1 levels are high in normal breast tissue and low in other normal epithelia. However, the absolute COL17A1 levels vary widely between various tumor types, as well as between various normal tissues. The different ratios between the numbers of basal and non-basal cells in different tissue types could partly account for that. Alternatively, or additionally, changes in the expression of one particular collagen gene could be compensated for by changes in the expression of other collagen genes.

In any case, our observations suggest that the expression of collagen XVII needs to be maintained at a tissue and cell type-specific normal level to prevent invasion. This thesis is supported by several previous observations. In keratinocytes, complete loss of *Col17a1* expression increases cell motility [36] and partial reduction of COL17A1 levels promotes undirected motility [37]. Conversely, collagen XVII expression is increased at the leading edge during wound healing [38] and at the invasive front of carcinomas [15], suggesting that it promotes motility. Consistently, COL17A1 supports directed migration by stabilizing actin bundles, which generates traction forces [37]. Together, this suggests that cancer cells may increase the invasive potential by either up- or downregulating collagen XVII expression.

## Conclusions

In conclusion, we identify breast cancer as the first type of cancer in which collagen XVII expression is underexpressed. We also find that the promoter methylation status correctly predicts whether collagen XVII is over- or underexpressed in various epithelial cancers. The underexpression in breast cancer is associated with increased invasion, while overexpression in other cancer types is also associated with increased invasion and metastasis. Functional studies are needed to mechanistically explain how collagen XVII overexpression affects cell motility, and its direction, and promotes tumor cell invasion and metastasis. However, our study has significant clinical implications, as it suggests that epigenetic targeting of *COL17A1* could represent a novel strategy to prevent metastasis in patients.

## Methods

### Clinical prognostic analyses

Data from 26 previously published breast cancer datasets were used to study the potential association between expression of each of the 44 collagen genes and distant metastasis-free survival, as described [23, 39]. Briefly, datasets were combined and statistical significance was determined according to a Cox proportional hazard model with 95% confidence interval (CI). Additional tests, according to Adjuvant! and the Nottingham Prognostic Index, were performed as described [17, 18]. Survival analyses were performed using a single clinical feature at a time, i.e., distant metastasis-free survival, recurrence-free survival, or overall survival, and by splitting the gene expression in tumors into below (low) and above (high) the median expression level, as described [19, 39]. Statistical analyses were performed using log-rank Mantel-Cox tests.

### Validation analyses

Validation analyses for *COL4A1*, *COL4A2*, and *COL17A1* gene expression in breast cancer were performed using 28 datasets, as previously described [19, 20]. In addition, the TCGA (The Cancer Genome Atlas) breast cancer and cervical cancer Illumina HiSeq RNA Seq V2 (RSEM-analyzed) datasets [21, 22] were used to compare *COL17A1* expression levels in matched normal control to breast and cervical cancer samples. Statistical analyses were carried out using *t* tests. Validation analyses for patient survival (distant metastasis-free survival, recurrence-free survival, and overall survival) were performed using probe 204636_at and the median as the cut-off between low and high expression, as described [23]. Log-rank tests were used to assess statistical significance.

### Immunohistochemistry

Paraffin-embedded breast cancer and cervical tissue microarrays (TMAs) were obtained from US Biomax Inc. (MD, USA). Samples were obtained under the Health Insurance Portability and Accountability Act (HIPAA)-approved protocols, in accordance with the approved guidelines and with informed consent from the donors. For the breast cancer analysis, only patients with ductal carcinoma pathology were included. However, no inclusion or exclusion criteria were applied based on treatments received. Cores were 5 μm thick and had a 1 mm diameter. TMAs were sectioned and stored at 4 °C until use. TMA slides were baked at 60 °C for 30 min, incubated in 100% xylene for 10 min for de-paraffinization, and incubated in ethanol series (100, 90, and 70%) and milliQ water for 10 min each for rehydration. For antigen retrieval using sodium citrate buffer (10 mM sodium citrate, 0.05% Tween-20, pH 6.0), slides were placed in a water bath, microwaved for 5 min (at P100/high), cooled for 5 min at room temperature (RT), and microwaved for 5 min. Slides were then cooled under tap water for 6 min, washed 2 × 5 min with PBS, and permeabilized in PBS/0.01% Triton-X for 10 min. Following 2 × 10 min PBS washes and 1 h incubation in blocking buffer (PBS/10% FBS), slides were incubated in blocking buffer with primary anti-collagen XVII antibody (clone 9G2, 1:100 dilution) [15] overnight at 4 °C, incubated at RT for 20 min, washed 2 × 5 min with PBS, blocked for endogenous peroxide in PBS/0.3% H_2_O_2_ for 10 min, washed 2 × 5 min with PBS, incubated with HRP-conjugated goat anti-mouse IgG (H + L) secondary antibody (Invitrogen, 62-6520) in blocking buffer for 1 h, followed by 2 × 10 min PBS washes. TMAs were then stained with 3,3′-diaminobenzidine (DAB) reagent (DBC859, Biocare Medical) and counter-stained with hematoxylin. Stepwise dehydration occurred in an ethanol series (70, 90, and 100%; 2 min each), followed by 9 min baking at 60 °C and incubation in 100% xylene for 2 min. Slides were mounted using Permount and dried overnight. Slides were imaged using Olympus Slide scanner VS120 (rm4026) and OlyVia software. Tissue sections were excluded from the analyses if chronic mastitis (breast), chronic inflammation/cervicitis, or cataplasia (cervix) was diagnosed or if more than 70% of the section was missing. Slides were independently scored by two individuals and in a blinded fashion. Clinical endpoints examined included pathology, age, grade, stage, tumor invasion, lymph node status, metastasis and estrogen (ER), progesterone (PR), and HER2 receptor status. Fisher’s exact tests were used for statistical analyses. In addition, we controlled for multiple testing by subjecting our analyses to a false discovery rate (FDR) of 5%, as previously described [40]. Where clinical data were missing for individual samples, these were excluded from the analyses involving the missing data, but included in analyses of other variables for which data were present. The clinicopathological details and standard prognostic variables of all patient samples subjected to immunohistochemistry are included in Additional file 1: Tables S2 and S3 for breast ductal carcinoma and cervical cancers, respectively.

### Allelic copy number variation and RNAseq analysis

Putative *COL17A1* allelic copy numbers were determined using Affymetrix Genome-Wide SNP6.0 Array datasets and GISTIC 2.0. RNAseq data, obtained from the TCGA breast cancer, cervical cancer, head and neck SCC, lung adenocarcinoma, and lung SCC Illumina HiSeq RNA Seq V2 (RSEM) datasets [21, 22, 41–43]. For each patient, copy number data and gene expression were combined and expression levels were plotted for each copy number category. Nonparametric Mann-Whitney *U* tests were used to compare differences.

### Promoter methylation analyses

For *COL17A1* promoter methylation analyses, Illumina Infinium HumanMethylation450 platform level 3 data were used from the respective TCGA cancer datasets [21, 22, 41–43]. For each sample, *β* values for all probes in the region from 400 bp upstream to 400 bp downstream of the *COL17A1* transcription start site (chromosome 10/hg19 genomic coordinate 105845638) were used (Additional file 2). These probes were: cg13553455 (10/105846002), cg08509991 (10/105845720), and cg13448625 (10/105845238). The probes are located at positions −364, −82, and +400 relative to the *COL17A1* transcription start site (TSS; see also Additional file 1: Figure S2). For each sample, the average of the *β* values was calculated and used. Data were not normally distributed, as determined by the D’Agostino and Pearson omnibus normality test *p* < 0.05. Therefore, nonparametric Mann-Whitney *U* tests were used to compare differences. RNAseq data from the respective TCGA datasets, as described above, were used to compare promoter methylation to gene expression. For linear regression, Spearman correlation analyses were used.

## Additional files

Additional file 1: Supplementary Material containing **Table S1** (Prognostic strength of misexpression of collagen genes in breast cancer), **Table S2** (Clinicopathological features of the breast cancer patients analyzed by immunohistochemistry), **Table S3** (Clinicopathological features of the cervical cancer patients analyzed by immunohistochemistry), **Figure S1** (H&E stained sections of corresponding samples shown in Fig. 2), **Figure S2** (Schematic of the promoter and 5’ end of the *COL17A1* gene), and Supplementary References. (PDF 1136 kb)

Additional file 2: *COL17A1* promoter methylation levels (β-values, TCGA Illumina Infinium Human Methylation 450). (XLSX 231 kb)

## Abbreviations

CI: Confidence interval
ECM: Extracellular matrix
ER: Estrogen receptor
FDR: False discovery rate
HR: Hazard ratio
IHC: Immunohistochemistry
PR: Progesterone receptor
SCC: Squamous cell carcinoma
TCGA: The Cancer Genome Atlas
