# Supplementary material for: In epithelial cancers, aberrant COL17A1 promoter methylation predicts its misexpression and increased invasion
Source: Clin Epigenetics. 2016 Nov 18;8:120. doi: 10.1186/s13148-016-0290-6 (PMC5116176; doi:10.1186/s13148-016-0290-6)
Supplement: Supplementary file 1 — Supplementary Material containing Table S1 (Prognostic strength of misexpression of collagen genes in breast cancer), Table S2 (Clinicopathological features of the breast cancer patients analyzed by immunohistochemistry), Table S3 (Clinicopathological features of the cervical cancer patients analyzed by immunohistochemistry), Figure S1 (H&E stained sections of corresponding samples shown in Fig. 2), Figure S2 (Schematic of the promoter and 5’ end of the COL17A1 gene), and Supplementary References. (PDF 1136 kb) [file 13148_2016_290_MOESM1_ESM.pdf]

Additional File 1: Supplementary Material

Table S1. Prognostic strength of misexpression of collagen genes in breast cancer

Gene [a]	No. of patients	HR (95% CI)	Prognostic strength p value	Adjuvant! Online [b] p value	Nottingham index [b] p value	Survival [c] p value
COL1A1	4176	1.04 (0.97-1.11)	0.2570	0.0895	0.0040	0.8796
COL1A2	4078	1.09 (1.02-1.16)	0.0086	0.0058	$9.01x10^{-5}$	0.6404
COL2A1	4077	1.04 (0.98-1.11)	0.2190	0.4464	0.2961	0.3380
COL3A1	3906	1.04 (0.98-1.11)	0.2190	0.0008	0.0001	0.4317
**COL4A1**	3925	1.25 (1.17-1.34)	$3.03x10^{-10}$	$3.94x10^{-6}$	0.0003	$1.71x10^{-5}$
**COL4A2**	4177	1.18 (1.11-1.25)	$8.11x10^{-10}$	0.0001	0.0018	0.0098
COL4A3	3609	0.91 (0.82-1.02)	0.0900	0.0009	0.0058	0.2711
COL4A4	3826	1.00 (0.93-1.07)	1.000	0.0161	0.0132	0.7605
COL4A5	3826	0.86 (0.81-0.92)	$4.57x10^{-6}$	0.0883	0.5144	0.0005
COL4A6	3825	0.93 (0.86-1.00)	0.0589	0.9200	0.4696	0.0057
COL5A1	4003	1.09 (1.02-1.16)	0.0086	0.0237	0.0006	0.1034
COL5A2	4078	1.08 (1.02-1.15)	0.0119	0.0130	0.0005	0.0925
COL5A3	3751	1.10 (1.03-1.17)	0.0034	0.0103	0.0002	0.2255
COL6A1	4127	1.02 (0.95-1.11)	0.6306	0.0741	0.0048	0.3263
COL6A2	3629	1.03 (0.97-1.11)	0.3971	0.0029	0.0067	0.4538
COL6A3	4177	1.05 (0.99-1.11)	0.0943	0.0790	0.0015	0.3380
COL6A5	1052	0.99 (0.86-1.13)	0.8939	0.5248	0.4144	0.4041
COL6A6	1545	0.80 (0.72-0.89)	$4.35x10^{-5}$	0.1516	0.1017	0.0010
COL7A1	3826	0.98 (0.91-1.05)	0.5920	0.5818	0.7821	0.7643
COL8A1	4078	1.04 (0.96-1.12)	0.3234	0.0383	0.0094	0.6404
COL8A2	3300	0.92 (0.86-0.99)	0.0201	0.9071	0.6913	0.2481
COL9A1	3826	1.07 (1.01-1.14)	0.0282	0.2339	0.0925	0.1282
COL9A2	3924	0.96 (0.90-1.02)	0.2026	0.3135	0.2763	0.3450
COL9A3	3925	1.02 (0.95-1.08)	0.5563	0.3450	0.1699	0.8796
COL10A1	3826	1.07 (1.01-1.15)	0.0407	0.0033	0.0021	0.0618
COL11A1	3826	1.13 (1.06-1.21)	0.0003	0.0869	0.1058	0.0415
COL11A2	3598	0.99 (0.91-1.07)	0.8195	0.6644	0.9105	0.2711
COL12A1	2269	1.05 (0.96-1.15)	0.2935	0.6181	0.2259	0.7199
COL13A1	2939	1.15 (1.04-1.27)	0.0061	0.4412	0.8771	0.0119
COL14A1	3709	0.90 (0.84-0.97)	0.0041	0.0594	0.1841	0.0015
COL15A1	3826	1.05 (0.99-1.12)	0.1211	0.0077	0.0283	0.6577
COL16A1	4176	0.94 (0.89-1.00)	0.0371	0.6270	0.3151	0.0522
**COL17A1**	3925	0.86 (0.81-0.92)	$4.57x10^{-6}$	0.0184	0.0434	0.0001
COL18A1	4127	1.08 (1.01-1.15)	0.0200	0.2976	0.0688	0.0355
COL19A1	3825	1.04 (0.98-1.11)	0.2190	0.3336	0.5479	0.1034
COL20A1	1915	1.00 (0.91-1.10)	1.000	0.1205	0.1547	0.3942
COL21A1	3923	0.97 (0.91-1.03)	0.3404	0.1376	0.0434	0.5144
COL22A1	1466	1.09 (0.97-1.24)	0.1697	0.7976	0.8749	0.0852
COL23A1	1967	0.97 (0.89-1.06)	0.5047	0.0486	0.0194	0.1979
COL24A1	1812	0.99 (0.91-1.09)	0.8383	0.8666	0.8134	0.8410
COL25A1	1722	1.01 (0.90-1.13)	0.8736	0.4467	0.2265	0.7004
COL26A1	1079	1.15 (0.96-1.37)	0.1235	0.7371	0.0790	0.8285
COL27A1	1722	0.85 (0.75-0.95)	0.0071	0.1076	0.1076	0.0061
COL28A1	1425	0.90 (0.79-1.03)	0.1195	0.2894	0.2709	0.8586

[a] *Bold-faced genes pass all four tests with p<0.05.*

[b] *Calculated as described in [1] and [2], respectively.*

[c] *Distant metastasis-free survival with patients split in low and high expression groups using the median expression level as the cut-off, as described [3]. P values based on log-rank Mantel-Cox tests.*

Table S2. Clinicopathological features of the breast cancer patients analyzed by immunohistochemistry

Clinicopathological variable	Number of patients (%)	Median (Range)
Gender		
Male	0 (0)	
Female	150 (100)	
Age		
<48	71 (47)	48 (27-75)
48-54	37 (25)	
>54	42 (28)	
Pathology		
Ductal carcinoma	150 (100)	
Grade		
1	3 (2)	
2	102 (68)	
3	10 (7)	
Stage		
I	6 (4)	
II	105 (70)	
III	33 (22)	
IV	6 (4)	
Tumor invasion		
T1	8 (5)	
T2	89 (59)	
T3	28 (19)	
T4	25 (17)	
Nodal status		
N0	98 (65)	
N1	38 (25)	
N2	12 (8)	
N3	2 (1)	
Metastasis		
M0	146 (97)	
M1	4 (3)	
Estrogen receptor status		
ER+	63 (42)	
ER-	78 (52)	
Progesterone receptor status		
PR+	44 (29)	
PR-	96 (64)	
HER2 status		
HER2-	32 (21)	
HER2+	108 (72)	

Table S3. Clinicopathological features of the cervical cancer patients analyzed by immunohistochemistry

Clinicopathological variable	Number of patients (%)	Median (Range)
Gender		
Male	0 (0)	
Female	361 (100)	
Age		
<48	199 (55)	46 (25-78)
48-54	94 (26)	
>54	68 (19)	
Pathology		
Squamous cell carcinoma	331 (92)	
Adenocarcinoma	27 (7)	
Adenosquamous carcinoma	3 (1)	
Grade		
1	56 (16)	
2	208 (58)	
2-3	8 (2)	
3	78 (22)	
Stage		
I	151 (42)	
II	34 (9)	
III	4 (1)	
IV	1 (0.3)	
Tumor invasion		
T1	240 (66)	
T2	103 (29)	
T3	10 (3)	
T4	0 (0)	
Nodal status		
N0	320 (89)	
N1	35 (10)	
N2	0 (0)	
N3	0 (0)	
Metastasis		
M0	355 (98)	
M1	2 (0.6)	

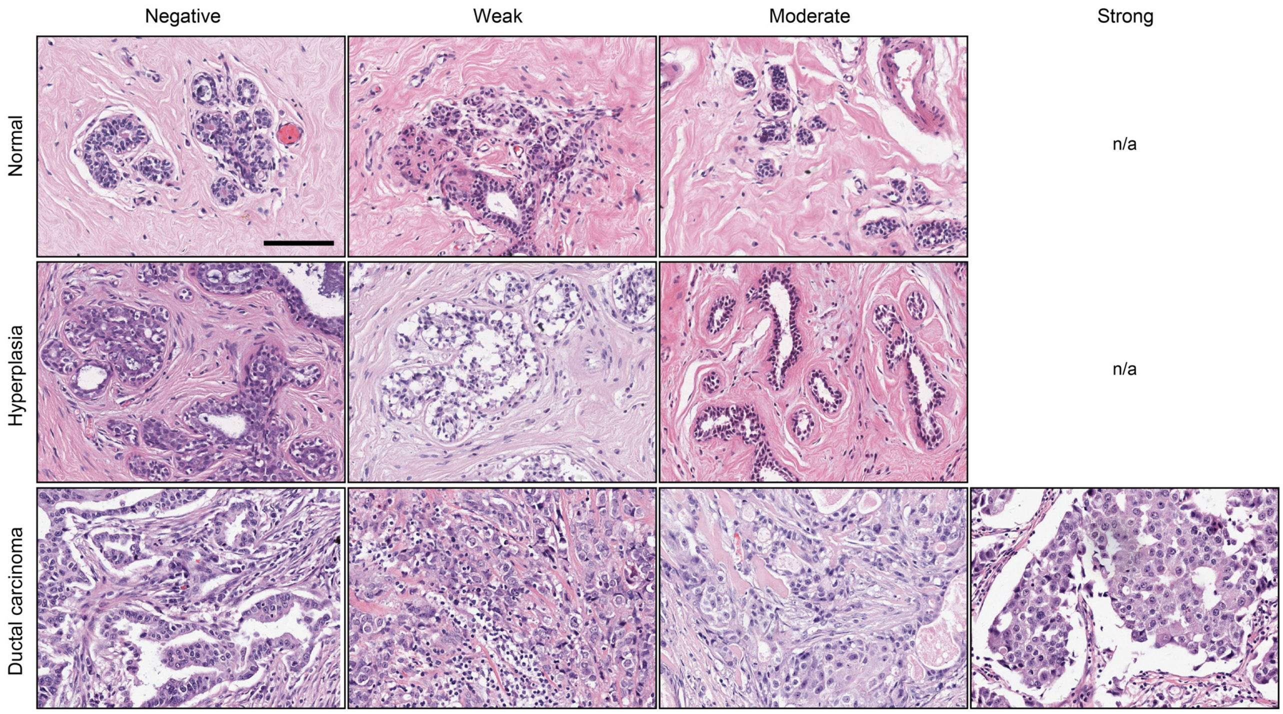

Figure S1.
H&E stained sections of corresponding samples shown in Figure 2. Scale bar, 100µm.

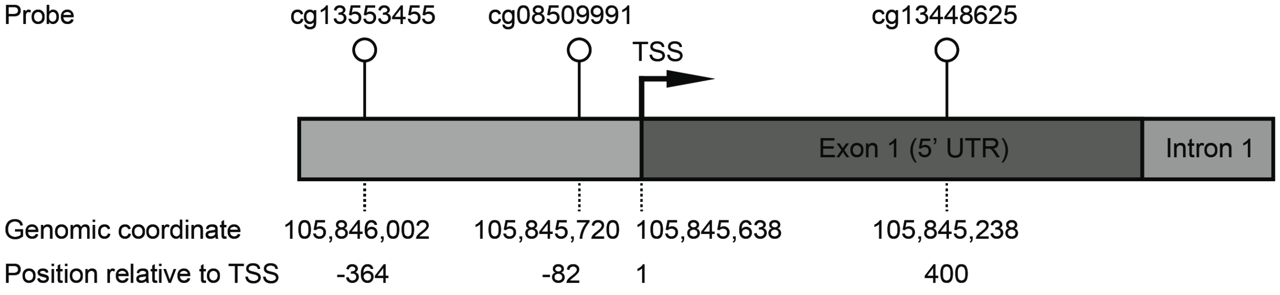

Figure S2. Schematic of the promoter and 5' end of the *COL17A1* gene.
The locations of the TCGA Illumina Infinium HumanMethylation450 probes, exon 1 and intron 1 are indicated relative to the transcription start site (TSS). The genomic coordinates correspond to the base pair numbers on chromosome 10, according to human reference genome hg19.

Supplementary References

1. Ravdin PM, Siminoff LA, Davis GJ, Mercer MB, Hewlett J, Gerson N, Parker HL. Computer program to assist in making decisions about adjuvant therapy for women with early breast cancer. J Clin Oncol. 2001; 19:980-91.
2. Galea MH, Blamey RW, Elston CE, Ellis IO. The Nottingham Prognostic Index in primary breast cancer. Breast Cancer Res Treat. 1992; 22:207-19.
3. Vaidyanathan S, Thangavelu PU, Duijf PH. Overexpression of Ran GTPase components regulating nuclear export, but not mitotic spindle assembly, marks chromosome instability and poor prognosis in breast cancer. Target Oncol. 2016; 11:677-86.
